# Global Health Preparedness Frameworks and Recombinant Vaccine Platforms: A Public Health Perspective on Regulations and System Readiness

**DOI:** 10.3390/vaccines14020144

**Published:** 2026-01-30

**Authors:** Luigi Russo, Leonardo Villani, Roberto Ieraci, Walter Ricciardi

**Affiliations:** 1 Section of Hygiene, University Department of Life Sciences and Public Health, Università Cattolica del Sacro Cuore, 00168 Rome, Italy; 2 UniCamillus-Saint Camillus International University of Health and Medical Sciences, Via di Sant’Alessandro, 00131 Rome, Italy; 3 Strategie Vaccinali, Regione Lazio, Via R. Raimondi Garibaldi, 7, 00145 Roma, Italy

**Keywords:** vaccination, preparedness, recombinant vaccine platforms, public health

## Abstract

**Background/objectives.** Emerging viral diseases represent an increasing threat to global health security, driven by environmental change, globalization, and intensified human–animal–environment interactions. The COVID-19 pandemic exposed critical weaknesses in preparedness systems but also demonstrated the transformative potential of recombinant vaccine technologies, which enable rapid, scalable, and safe responses to novel pathogens. We aim to examine the role of recombinant vaccine platforms in the management of emerging viral diseases, emphasizing their contribution to health system preparedness and exploring strategies for their integration into preparedness frameworks. **Methods.** We synthesized the current evidence on recombinant vaccine platforms (viral vector, protein subunit, DNA, and mRNA) through a targeted review of the scientific literature, regulatory documents, and global health policy reports. Drawing from experiences like COVID-19 (mRNA vaccines) and Ebola (rVSV-ZEBOV), we analyzed the advantages, challenges, and lessons from initiatives such as the CEPI, BARDA, HERA, and WHO frameworks. **Results.** Recombinant vaccine platforms offer significant advantages for epidemic preparedness through rapid adaptability, standardized production, and strong safety profiles. Nonetheless, challenges remain in manufacturing scalability, cold-chain logistics, regulatory harmonization, and equitable global access. Global initiatives such as the CEPI, WHO-led programs, BARDA, and regional manufacturing networks exemplify this collaborative approach, while regulatory mechanisms have proven to be essential to timely vaccine deployment. **Conclusions.** Recombinant vaccines have redefined preparedness by coupling scientific innovation with operational agility. Strengthening global coordination, regional production capacity, and public trust is essential to ensure that technological progress translates into equitable and effective public health impacts.

## 1. Introduction

Emerging viral diseases represent one of the most pressing challenges to global health security in the twenty-first century [1]. The increasing frequency of outbreaks, such as those caused by Ebola, Zika, Nipah, and, most notably, SARS-CoV-2 [2], reflects complex interactions between the environment and climate change [3], globalization, urbanization, and increased human mobility, as well as the intensified human–animal–environment interactions, with zoonotic spillover events becoming increasingly frequent [4]. These events have demonstrated how rapidly a novel pathogen can disrupt societies, overwhelm health systems, and expose weaknesses in public health preparedness.

Preparedness is defined by the World Health Organization (WHO) as the capacity of health systems and societies to anticipate, respond to, and recover from public health emergencies [5]. In this context, investing in preparedness and innovative tools to quickly respond to these kinds of health emergencies is crucial. New technologies, such as recombinant vaccines and immunotherapy strategies, have substantially strengthened epidemic preparedness and response capacities by enabling rapid development, scalable manufacturing, and targeted deployment of countermeasures against novel pathogens [6,7].

Recombinant platforms contain different innovative technologies, most of them able to ensure quick responses and fast development, including viral vector vaccines (such as recombinant adenovirus), nucleic acid-based vaccines (mRNA and DNA vaccines, often delivered via lipid nanoparticles), and recombinant protein subunit vaccines [8].

Beyond their scientific value, these technologies have profound implications for public healthcare systems. They demand new models of governance, supply chain readiness, manufacturing infrastructure, and risk communication [8]. The COVID-19 pandemic has demonstrated both the potential of recombinant technologies to accelerate vaccine development and the persistent inequities in access and distribution that limit their global impact [9].

In this context, we aim to describe the role of recombinant vaccine platforms in the management of emerging viral diseases as viruses currently represent the most frequent cause of large-scale epidemics and pandemics and the primary target of recombinant vaccine platform development, emphasizing their contribution to health system preparedness and how health systems can integrate these innovations into preparedness frameworks, from a public health perspective.

## 2. Recombinant Vaccine Technologies: Overview and Public Health Relevance

Recombinant vaccine technologies represent a major advancement in modern vaccinology and epidemic preparedness [10]. Unlike traditional live attenuated or inactivated vaccines, recombinant vaccines employ genetic engineering to express specific antigens that are capable of evoking protective immune responses without handling the pathogen itself [11]. This strategy ensures high safety, scalability, and adaptability, all crucial for rapid responses to emerging infectious diseases [8]. At the same time, it introduces challenges that require streamlined regulatory frameworks, global coordination, and equitable access mechanisms.

Over the past two decades, recombinant platforms have diversified, each with distinct advantages and limitations. The key types include recombinant protein subunit vaccines [12], which express antigenic proteins in host cells such as yeast or mammalian systems, and virus-like particle (VLP) vaccines [13], exemplified by the HPV vaccine, which form self-assembling non-replicating structures that mimic native viruses. Additional categories include recombinant viral vector vaccines [14], DNA vaccines (mostly in veterinary medicine; only one vaccine approved for use in humans) [15,16,17], and mRNA vaccines [18].

These platforms demonstrate how recombinant technologies can balance safety, efficacy, and adaptability to address evolving public health needs and are summarized in Table 1.

### 2.1. Applications of Recombinant Platforms and the Recent Role of mRNA Platforms

Recombinant vaccine platforms have successfully established their public health relevance in the last twenty years, leading to the development of vaccines against key pathogens. Examples include the rVSV-ZEBOV vaccine deployed during Ebola outbreaks [25], which has demonstrated high efficacy in containing the outbreaks and in reducing mortality, including in already infected patients [26,27]. Even more established is the role of recombinant protein vaccines for hepatitis B and HPV, with decades of real-world use, efficacy and safety [28].

More recently, mRNA-based vaccine platforms have made an impact in modern vaccinology, with the global success of mRNA vaccines during the COVID-19 pandemic providing evidence of their effectiveness, safety, and production agility. Building on this success, mRNA technologies are now being applied to other major viral threats. A recent example is their use against Orthoflaviviruses, a group that includes dengue, Zika, yellow fever, and tick-borne encephalitis viruses, which are responsible for significant and recurrent global outbreaks [25]. These platforms allow for the precise engineering of viral antigens, such as masking enhancement-prone epitopes or incorporating non-structural proteins like NS1, to improve both the breadth and safety of the immune response [25] and allow adaptation to emerging variants through their modular design. All these platforms can induce rapid, robust, and durable immune responses, which, combined with the scalability and rapid manufacturing capacity, particularly of mRNA systems, position recombinant platforms as a cornerstone for future epidemic preparedness and rapid-response vaccine development.

### 2.2. Advantages and Limitations for Public Health Preparedness

From a public health perspective, recombinant vaccines offer several key advantages that enhance epidemic preparedness and enable rapid responses to emerging threats. Recombinant platforms allow for rapid design and production once the genetic sequence of a pathogen is known, as demonstrated during the COVID-19 pandemic by mRNA vaccines [29,30], the Ebola outbreaks by viral vector vaccines [26,27] or seasonal influenza by protein subunit vaccines [20]. Moreover, the modular nature of recombinant technologies [29] enables quick adaptation to emerging variants or new pathogens by altering the genetic sequence encoding the antigen. They do not contain live pathogens, eliminating the risks of reversion to virulence and contamination [31], while production can be highly standardized, allowing for consistent quality and easier scale-up to meet demand [30].

These attributes align closely with the principles of epidemic preparedness, anticipation, adaptability, and rapid response.

Despite their advantages, challenges for health system implementation persist. Recombinant vaccine platforms are not inherently “plug-and-play” systems as their successful deployment critically depends on prior knowledge of suitable antigenic targets. Protective antigens must be identified in advance, be sufficiently conserved to counteract antigenic variability, and be correctly folded to preserve conformational and post-translationally modified epitopes that are essential for effective immune recognition.

As a result, recombinant vaccine development remains highly knowledge-intensive and requires substantial upfront investment in basic research, structural biology, and immunology before accelerated manufacturing timelines can be realistically achieved.

Moreover, the manufacturing complexity of some platforms (e.g., viral vectors or protein subunit vaccines), and their related costs, can limit scalability [12], while others (notably mRNA vaccines) require stringent cold-chain logistics [11], posing challenges for distribution, especially in resource-limited settings. These kinds of vaccines tend to be less immunogenic than live-attenuated or whole-pathogen vaccines, often requiring adjuvants or multiple doses to achieve adequate protection [32]. Finally, the development of a novel recombinant technology is associated with significant financial, regulatory, and temporal costs. The generation of a new vaccine, including antigen discovery, preclinical development, clinical trials, and regulatory approval, requires heavy investments and may face lengthy and complex regulatory pathways if those are not already standardized and ready for emergencies, highlighting the lack of preparedness for major epidemic events [33].

Future emerging pathogens may not exhibit the same vulnerability to antibody-mediated protection. In such scenarios, alternative vaccine strategies, including inactivated or live-attenuated vaccines, may still play a complementary role by providing partial or non-sterilizing immunity that is capable of reducing disease severity and prolonging survival while more targeted recombinant solutions are developed.

Another concern relates to public trust [34]. Since these technologies are often developed rapidly in emergency contexts, long-term data are limited, which can lead to hesitancy and a lack of confidence, as commonly observed in these contexts [35,36]. These considerations underscore that scientific innovation alone does not guarantee public health impact as the successful integration of recombinant vaccines into epidemic preparedness plans depends on systemic readiness and community engagement.

## 3. Health System Preparedness for Emerging Viral Diseases

### 3.1. Preparedness: A State of the Art

Health system preparedness is the process of planning, organizing, equipping, training, exercising, evaluating, and improving activities to ensure effective coordination and response to public health emergencies [5]. Preparedness is an integral component of the International Health Regulations (IHR), first published in 2005 and signed by all 196 countries adhering to the WHO. The IHR framework requires all state parties to establish and sustain preparedness capacities and has been amended multiple times in 2014, 2022 and 2024, introducing in 2016 an external evaluation mechanism, the Joint External Evaluation (JEE) [37]. Moreover, after the COVID-19 pandemic, during the 78th World Health Assembly, the 2025 WHO Pandemic Agreement [38,39] was ratified, introducing several changes under the umbrella of a One Health approach, including the obligation to develop national plans for pandemic prevention and surveillance, to mitigate drivers of zoonotic spillover (e.g., climate change), and to ensure equitable access to pandemic countermeasures [38,39].

These measures are critical due to the unique challenges posed by emerging viral diseases, including their unpredictable nature, rapid transmissibility, and potential for international spread [40]. The past two decades have furnished several examples [41], from the 2003 SARS outbreak to the 2014–2016 Ebola epidemic and the recent COVID-19 pandemic, highlighting that no healthcare system is immune to the disruptive effects of novel pathogens [42]. This burden extends well beyond morbidity and mortality, affecting economic stability, social cohesion, and ultimately the resilience of healthcare systems [37].

An effective strategy to face these outbreaks is to improve preparedness strategies and strengthen public health responses. Preparedness involves multiple interconnected domains, including surveillance and early detection [1,43], laboratory and diagnostic capacity [1,43], emergency response coordination [44], health workforce readiness and continuous training [1,42], supply chain and infrastructure resilience, including vaccine storage and cold-chain logistics [1,43,45], and risk communication and community engagement [46].

Moreover, in addition to vaccines, preparedness strategies consider the role of repurposed pathogen-directed therapeutics as non-vaccine medical countermeasures that include antivirals and monoclonal antibodies, which can be rapidly deployed during the early stages of outbreaks [47,48]. These interventions may be particularly relevant in settings characterized by limited vaccine acceptance or delayed vaccine availability, providing a complementary layer of protection while vaccine-based immunity is established [47].

Preparedness, however, revolves around many possible threats that may disrupt health systems, most notably respiratory and contact-based viruses with pandemic potential (e.g., coronaviruses, Ebola, influenza, and Disease X), vector-borne or animal-reservoir viruses with epidemic potential (e.g., dengue, West Nile virus, and chikungunya) [1], antimicrobial resistance (AMR), and armed conflict-related threats and Chemical, Biological, Radiological and Nuclear (CBRN) threats, both accidental and intentional [49].

### 3.2. Proactive and Reactive Preparedness: Lesson Learned from the COVID-19 Pandemic and Current Existing Frameworks

Traditional models of epidemic response have often been reactive, mobilizing resources only after an outbreak occurs. The COVID-19 pandemic has emphasized that preparedness cannot rely solely on emergency response mechanisms but must be embedded in health system functions, requiring sustained investment in surveillance, regulatory frameworks, and flexible manufacturing capabilities [43,50,51]. In this sense, recombinant vaccines enable a paradigm shift toward proactive preparedness [42] as they are not just biomedical innovations but represent strategic tools for rapid response [50,52].

Several international initiatives emerged to strengthen global capacities for epidemic and pandemic response [51] well before the COVID-19 pandemic, focusing on early research coordination, vaccine platform development, and equitable access mechanisms. Operating across complementary domains, these initiatives may be state-funded (e.g., the BARDA [53] in the United States), globally coordinated (e.g., the WHO R&D Blueprint) [5], or targeted toward low- and middle-income countries (e.g., the PAVM). The most relevant initiatives are summarized in Table 2.

Despite the proliferation of global coalitions and preparedness initiatives, most international efforts for pandemic response continue to rely on the WHO as the primary authority for response and coordination. While this central role has historically ensured global legitimacy and technical consistency, recent geopolitical tensions and resource constraints have raised concerns regarding the sustainability and effectiveness of a predominantly WHO-centered model. As shown in Table 2, multiple initiatives that operate outside the WHO through different governance models, mandates, and levels of autonomy are being financed.

Recombinant technologies have also underscored the importance of regulatory preparedness, defined as the ability of national authorities to evaluate and authorize novel biomedical products swiftly and transparently [54]. The experience of the COVID-19 pandemic offers, again, valuable lessons in this regard [9]. For example, in the United States, the Food and Drug Administration (FDA) utilized the Emergency Use Authorization (EUA) mechanism, which allowed the temporary use of COVID-19 vaccines and their rapid deployment during the emergency [55,56].

As per the European Union, a more complex mechanism was put in place. First, the EU implemented a joint procurement mechanism that ensured coordinated and equitable access across all Member States. Through the EU Vaccines Strategy of June 2020 [57], the European Commission, acting on behalf of Member States, negotiated Advance Purchase Agreements (APAs) [58] with vaccine developers using EU funds under the Emergency Support Instrument [59]. These agreements secured early access to promising vaccine candidates by providing upfront financing in exchange for guaranteed supply once regulatory approval was granted. Distribution followed a population-based allocation, ensuring fairness and solidarity among Member States under the oversight of a Steering Board representing all EU countries. Moreover, the European Medicines Agency (EMA) implemented a rolling review process and granted Conditional Marketing Authorization (CMA) [60]. The rolling review enabled the EMA to assess data as they became available, significantly accelerating the evaluation timeline [55,61]. This process led to the approval of five COVID-19 vaccines through rolling review followed by CMA, namely Comirnaty (Pfizer-BioNTech, New York City, NY, USA/Mainz, Germany), Spikevax (Moderna, Cambridge, MA, USA), Vaxzevria (AstraZeneca, Cambridge, UK), the Janssen COVID-19 Vaccine (Johnson & Johnson, New Brunswick, NJ, USA), and Nuvaxovid (Novavax, Gaithersburg, MD, USA).

This collective approach avoided internal competition for limited supplies and leveraged the EU’s market power to obtain favorable conditions from manufacturers.

In both regions, rigorous scientific and regulatory standards were maintained without compromising safety [61]; however, the expedited pathways were crucial for a timely public health response. Streamlining these processes while preserving safety and public trust remains a central challenge for refining preparedness in future major health emergencies [52].

Moreover, effective preparedness requires that countries maintain reliable and diversified supply chains for essential medical countermeasures [62]. The COVID-19 pandemic highlighted critical vulnerabilities associated with overreliance on external manufacturing sources as delays in access to personal protective equipment, oxygen concentrators, and other essential supplies hampered timely response efforts in many settings [63]. Strengthening domestic and regional production capacity, together with supply chain diversification and pre-agreed procurement mechanisms, represents a key component of preparedness and resilience, complementing investments in vaccine platforms and regulatory readiness.

### 3.3. The European Example: The Health Emergency Preparedness and Response Authority (HERA)

The European Union represents a virtuous example of efforts aimed at ensuring a coordinated and effective response to emergencies through the adoption of several strategies and regulations. The first step was the approval of Regulation (EU) 2022/2371 [64] on serious cross-border threats to health to create a stronger mandate for coordination and cooperation for a more effective response to serious cross-border health threats.

Moreover, the EU has strengthened its health crisis preparedness through the establishment of the Health Emergency Preparedness and Response Authority (HERA) within the Directorate-General for Health and Food Safety (DG SANTE) [65]. Functioning as the EU’s central “health security watchtower,” the HERA coordinates preparedness, threat assessment, and response capacity in relation to medical countermeasures (MCMs), including vaccines, therapeutics, diagnostics, and personal protective equipment [66].

In this framework, the EU Medical Countermeasures Strategy (2025) [67] was recently published, which positions MCMs as strategic assets that are essential for Europe’s security and resilience. Building on the lessons from COVID-19, the strategy promotes a One Health all-hazards approach—from early threat detection and R&D investment to scalable production and equitable access. It introduces initiatives such as the Medical Countermeasures Accelerator to streamline innovation funding, the RAMP-UP manufacturing partnership to secure rapid production capacity, and the EU List of Priority Threats and MCMs to guide preparedness investments. Together, these efforts have the objective to strengthen European Union countries’ capability of responding to future pandemics and health emergencies.

## 4. Integrating Recombinant Platforms into Public Health Preparedness Plans

The integration of recombinant vaccine platforms into national and global preparedness frameworks represents a critical evolution in the way health systems anticipate and manage emerging viral threats. Preparedness is no longer confined to surveillance and emergency response; as summarized in Table 3, preparedness depends on coordinated actions across multiple interdependent domains, and addressing these domains systematically is essential to ensure that recombinant platforms can be rapidly and effectively deployed during emerging health emergencies [68,69].

Preparedness planning should prioritize platforms with demonstrated safety, immunogenicity, and manufacturability as not all recombinant platforms are equally suited for rapid outbreak response. Platforms with prior regulatory approval or advanced clinical data enable faster antigen replacement without restarting full development programs. Antigen design must rely on validated targets with preserved structural integrity and relevant post-translational modifications [70].

Accelerated regulatory pathways should be defined in advance and linked to specific platforms. Rolling reviews, emergency authorizations, and conditional approvals are most effective when prior platform data can be reused and must be pre-established and coordinated across jurisdictions [71]. Regulatory reliance and work-sharing mechanisms between agencies reduce duplication and accelerate decision-making [61].

Manufacturing scalability and readiness should be pursued through the assessment of production capacity and readiness for technology transfer. Platforms supported by standardized modular manufacturing processes facilitate faster scale-up. Regional hubs should be identified and maintained to strengthen manufacturing capability, reducing dependence on a limited number of global suppliers and enhancing resilience against supply disruptions [72].

Recombinant platforms differ substantially in their logistical requirements. End-to-end logistics, including cold-chain, packaging, and distribution constraints, must be mapped for each platform in advance, as well as developing contingency strategies for temperature-sensitive products [73,74]. Temperature-sensitive platforms require contingency planning for storage, transport, and last-mile delivery, particularly in resource-limited settings.

Stockpiling strategies should distinguish between bulk antigen, intermediates, and finished products depending on platform stability [75,76]. Deployment protocols must define prioritization criteria target populations, and distribution mechanisms before emergencies occur [71].

Since safety surveillance and pharmacovigilance are indispensable for sustaining public trust, robust platform-specific safety systems for real-time monitoring of adverse events, coupled with mechanisms for long-term safety follow-up, must be integrated into routine surveillance infrastructure. Interoperable pharmacovigilance systems enable rapid signal detection when vaccines are deployed at scale [53].

Health systems should incorporate platform-specific requirements into immunization programs, including storage, preparation, administration, and risk communication. Training the healthcare workforce should reflect differences across vaccine technologies, their administration and usage [69].

Long-term funding mechanisms are required to sustain platform readiness between emergencies. Investments should cover research and development, manufacturing maintenance, and rapid scale-up capacity, supported by public–private partnerships [71].

Mechanisms for equitable access, including dose-sharing arrangements, technology transfer, and regional production, must be incorporated in preparedness plans. Aligning them with global and regional equity initiatives strengthens collective response capacity and mitigates disparities [50].

Preparedness performance should be assessed using predefined indicators for development timelines, production output, deployment speed, and coverage, enabling adaptive updates to preparedness plans [71].

In this context, pros and cons for each platform must be acknowledged. mRNA vaccines show clear advantages in domains related to platform adaptability and maturity, regulatory alignment, and emergency regulatory pathways, where prior platform data can support accelerated review processes. However, they present critical limitations in the supply chain and logistics due to stringent cold-chain requirements, in stockpiling strategies given limited long-term stability, and financial constraints related to the high costs of their development. Recombinant protein subunit vaccines are particularly well suited for manufacturing scalability, supply chain management, stockpiling, and equity and access. Their limitations primarily relate to slower development timelines and lower intrinsic immunogenicity, often requiring adjuvants and multiple doses. Virus-like particle vaccines share many advantages with protein subunit platforms, including strong safety profiles, ease of integration into immunization programs, and simplified safety surveillance. Their main constraints are manufacturing complexity and higher production costs, which may limit rapid scale-up during large outbreaks. Recombinant viral vector vaccines perform well in domains related to rapid deployment, induction of cellular immunity, and emergency response, particularly where single-dose strategies are advantageous. Nonetheless, their suitability is constrained by pre-existing vector immunity, platform-specific safety considerations, and more complex regulatory evaluation.

## 5. Future Directions and Policy Implications

The integration of recombinant vaccine technologies into preparedness frameworks marks a transformative step toward a more anticipatory model of global health security. Future efforts should focus on consolidating this shift through sustained investment in platform technologies, streamlined regulatory pathways, and permanent mechanisms for global coordination and data sharing. Strengthening regional manufacturing capacity and technology transfer will be essential to ensure equitable access and to reduce dependence on a limited number of producers. Likewise, embedding regulatory preparedness within national health systems, through pre-established emergency pathways, reliance models through which regulatory authorities may use evaluations conducted by other trusted agencies to facilitate decision-making, and continuous training, will help to translate scientific innovation into timely public health action. Beyond technical and regulatory progress, the long-term success of recombinant technologies will depend on public trust and transparent communication. Lessons from the COVID-19 pandemic underscore that scientific excellence alone is insufficient without societal confidence and equitable benefit sharing. Building resilient preparedness frameworks therefore requires a holistic approach that integrates innovation, governance, and equity. If effectively implemented, recombinant technologies can serve as both a scientific and structural cornerstone for a new era of proactive globally coordinated pandemic preparedness. The new preparedness paradigm is thus one that combines scientific agility with social responsibility, ensuring that the benefits of innovation are shared globally and equally.

## 6. Conclusions

Integrating recombinant platforms into preparedness frameworks marks a transformative shift toward anticipatory global health security. Future efforts should prioritize sustained investments in platform technologies, streamlined regulatory pathways, and permanent global coordination mechanisms. Strengthening regional manufacturing and technology transfer will ensure equitable access and reduce dependencies. Embedding regulatory preparedness in national systems through pre-established emergency pathways and reliance models will accelerate public health action. A holistic approach combining innovation, governance, equity, and transparent communication is needed to build resilient frameworks for proactive pandemic response.

## Figures and Tables

**Table 1:** Main recombinant vaccine types.

Vaccine Type	Technology Principle	Representative Examples	Advantages	Disadvantages	Public Health Relevance
Recombinant protein subunit vaccines [12,13]	Expression of antigenic proteins in host cells (e.g., yeast, bacteria, insects, mammalian cells)	Novavax COVID-19 vaccine [19]; Recombinant influenza vaccines [20]	High safety, no risk of infection, scalable production	Often less immunogenic, requires adjuvant, may need multiple doses	Safe and well-characterized
Virus-like particle (VLP) vaccines [21]	Self-assembling non-replicating structures mimicking viral morphology	HPV [22], Candidate Zika VLP vaccines [23]	Highly immunogenic, mimics native virus structure, no genetic material	Complex manufacturing, stability issues, cost	High immunogenicity; flexible production platforms
Recombinant viral vector vaccines [14]	Use of replication-defective viruses as carriers of target antigens	rVSV-ZEBOV (Ebola) [24], ChAdOx1 (AstraZeneca COVID-19) [19]	Induces strong cellular and humoral immunity, mimics natural infection	Pre-existing vector immunity, potential vector-related adverse effects	Suitable for rapid deployment; strong cellular immunity
DNA vaccines ^a^ [15]	Plasmid DNA encoding antigen delivered to host cells for in situ expression	Used in veterinary medicine. Only one approved human DNA vaccine exists (ZyCoV-D) ^b^ [16,17]	Induces both humoral and cellular immunity, stable, easy to produce	Limited immunogenicity in humans, theoretical risk of integration	Application for veterinary medicine in a OneHealth approach
mRNA vaccines ^a^ [18]	Synthetic mRNA encoding antigen delivered (often in lipid nanoparticles)	COVID-19 mRNA vaccines [19]	Rapid development, no risk of genome integration, strong immune response	Requires cold chain, mRNA instability, reactogenicity	Flexible and adaptable platform for vaccine design; adaptable to new variants; rapidly scalable production

^a^ = Although nucleic acid-based vaccines are not “recombinant” in the classical sense, they are part of the same technological continuum. ^b^ = approved in India.

**Table 2:** Relevant initiatives for epidemic preparedness.

Initiative/Organization	Year of Establishment	Core Mission/Focus	Relevance to Recombinant Vaccine Platforms	Link
Pandemic Fund (World Bank)	2022	Provides financial support for countries to strengthen pandemic prevention, preparedness, and response capacities.	Enables sustainable investment in manufacturing and readiness for recombinant vaccine platforms.	https://www.thepandemicfund.org (accessed on 26 January 2026)
The Health Emergency Preparedness and Response Authority’s (HERA)	2021	HERA is responsible for EU health preparedness and crisis response. In the case of an emergency, HERA will oversee the development, production and distribution of medicines, vaccines and other medical countermeasures (MCMs).	Funding R&D of vaccines, supporting research, manufacture, purchasing, and distribution of MCMs in the event of a health emergency	https://commission.europa.eu/about/departments-and-executive-agencies/health-emergency-preparedness-and-response-authority_en (accessed on 26 January 2026)
WHO Hub for Pandemic and Epidemic Intelligence	2021	Strengthens global intelligence for early detection, risk assessment, and data sharing on outbreaks.	Supports evidence-based prioritization of vaccine platform activation during emerging threats.	https://pandemichub.who.int (accessed on 26 January 2026)
Africa CDC—Partnership for African Vaccine Manufacturing (PAVM)	2021	Builds sustainable vaccine production capacity across Africa to improve regional self-sufficiency.	Focuses on technology transfer and local production of recombinant and mRNA vaccines.	https://africacdc.org/download/partnerships-for-african-vaccine-manufacturing-pavm-framework-for-action/ (accessed on 26 January 2026)
Coalition for Epidemic Preparedness Innovations (CEPI)	2017	Accelerates vaccine development against emerging infectious diseases and enables equitable access during outbreaks.	Major funder of recombinant and mRNA vaccine R&D (e.g., COVID-19, Lassa, Nipah); promotes rapid platform adaptation.	https://cepi.net (accessed on 26 January 2026)
WHO R&D Blueprint	2015	Defines global priorities and accelerates R&D for high-threat pathogens lacking countermeasures.	Provides global guidance for platform technologies and harmonized clinical trial designs.	https://iris.who.int/server/api/core/bitstreams/b03999bf-4ff3-4827-8309-9c368bb4bfa1/content (accessed on 26 January 2026)
The Global Health Security Agenda (GHSA)	2014	Strengthens national and global capacity to prevent, detect, and respond to infectious disease threats.	Promotes integration of vaccine R&D into broader preparedness frameworks and health security plans.	https://globalhealthsecurityagenda.org (accessed on 26 January 2026)
GLOPID-R (Global Research Collaboration for Infectious Disease Preparedness)	2013	Coordinates global research funding and collaboration for emerging infectious diseases.	Facilitates data sharing and alignment of research priorities, supporting recombinant vaccine trials and rapid response studies.	https://www.glopid-r.org (accessed on 26 January 2026)
BARDA (Biomedical Advanced Research and Development Authority, USA)	2006	In the context of the Administration for Strategic Preparedness and Response (ASPR), the US agency that leads the nation’s medical and public health preparedness, BARDA supports advanced development and procurement of medical countermeasures for health emergencies.	Major investor in recombinant and viral vector vaccines; facilitates public–private partnerships and rapid scale-up.	https://aspr.hhs.gov/AboutASPR/ProgramOffices/BARDA/Pages/default.aspx (accessed on 26 January 2026)
Gavi, the Vaccine Alliance	2000	Improves equitable access to vaccines in low- and middle-income countries through financing and partnerships.	Key to deployment and equitable distribution of recombinant and mRNA vaccines during pandemics.	https://www.gavi.org (accessed on 26 January 2026)

**Table 3:** Proposed domains for inclusion of recombinant vaccine platforms in preparedness frameworks.

Domain	Action(s)
Platform adaptability and maturity	Assess whether the platform can be rapidly customized to emerging pathogens and whether it has a proven record of safety, immunogenicity, and manufacturability. Platforms with prior regulatory approval or clinical validation should be prioritized.
Regulatory alignment and harmonization	Develop coordinated regulatory pathways that enable accelerated review and emergency authorization across regions. Encourage data sharing, reliance mechanisms, and international harmonization to speed up deployment.
Manufacturing scalability and readiness	Evaluate existing production capacity, potential for rapid scale-up, and readiness of facilities for technology transfer. Strengthen regional manufacturing hubs to reduce dependency on limited suppliers.
Supply chain and logistics	Map out end-to-end logistics, including cold-chain, packaging, and distribution requirements. Build contingency plans for temperature-sensitive products, such as mRNA or vector-based vaccines.
Stockpiling and deployment protocols	Establish pre-agreed stockpiling strategies and deployment frameworks. Define prioritization criteria, target populations, and mechanisms for rapid mobilization during outbreaks
Safety surveillance and pharmacovigilance	Implement robust systems for real-time monitoring of adverse events and long-term safety. Promote international data sharing and interoperability between pharmacovigilance networks.
Program planning and workforce training	Integrate recombinant platforms into national immunization programs. Provide targeted training for healthcare workers for specific vaccine types, emphasizing handling, administration, and communication with the public.
Financing and sustainable investment	Secure dedicated funding for R&D, manufacturing scale-up, and emergency deployment. Encourage public–private partnerships and long-term investment in platform-based vaccine innovation.
Equity and access	Design mechanisms to ensure equitable access to recombinant vaccines across countries and populations. Align with global initiatives for dose sharing, technology transfer, and local production.
Monitoring and evaluation	Define indicators for measuring program performance, vaccine coverage, and timeliness. Establish systems for real-time data collection, feedback, and adaptive policy updates

## Data Availability

Data are contained within the article.
